# Placentitis Occurring Twice in the Same Woman

**Published:** 1848-10

**Authors:** 


*Placentitis Occurring Twice in the same Woman.*—Dr. Van Hengel attended a woman, thirty-three years of age, who was delivered of a child, of whose death there had been distinct signs three weeks previously. The fœtal portion of the placenta had degenerated into a greyish-white colour ; on the uterine surface it was still spongy and porous in several spots. Two years afterwards the woman was again near the time of her delivery. She stated that between the seventh and eighth month she had had a slight attack of fever, after which she was seized with severe pain in the right side of the abdomen, in which part she felt as if there was a weight lying within her; at the same time she suffered from thirst, sleeplessness, headache, and loss of appetite. Subsequently she was troubled at various times with bloody, watery, and purulent discharges from the vagina. The pulsation of the fœtal heart could not be heard, nor could any movements of the child be felt by the mother or her medical attendant ; and at the same time she complained of nausea and a sensation of cold in the belly. Some days after, she was delivered very quickly of a child which appeared to have been long dead. The placenta was circular, curled inwards at the edges, greyish-yellow in colour. On the fœtal surface it was dark-brown or almost black ; and it was so indurated as not to bend when held out by one point.—*Ibid.*

